# Evolution of Peripheral Retinal Perfusion in Sickle Cell Retinopathy During Severe Vaso-Occlusive Crises: A Report of Two Cases

**DOI:** 10.7759/cureus.117897

**Published:** 2026-10-14

**Authors:** Elena Missa, Nikola Filipovic, Ismael Daoudi, Iris Païdassi, Laurence Postelmans, Florence Rasquin, Sina Hakami

**Affiliations:** 1 Ophthalmology, Hôpital Érasme, Brussels, BEL; 2 Ophthalmology, Centre Hospitalier Universitaire de Saint-Pierre, Brussels, BEL; 3 Ophthalmology, Hôpital Erasme, Brussels, BEL; 4 Ophthalmology, Centre Hospitalier Universitaire Brugmann, Brussels, BEL

**Keywords:** exchange transfusion, fluorescein angiography, peripheral retinal ischemia, retinal nonperfusion, retinal reperfusion, sickle cell retinopathy, vaso-occlusive crisis

## Abstract

Sickle cell retinopathy (SCR) is characterized by retinal vaso-occlusion and peripheral ischemia. We describe two patients with homozygous sickle cell disease (HbSS) who presented with extensive peripheral retinal nonperfusion during severe vaso-occlusive crises. Multimodal retinal imaging was performed during the acute phase and after systemic stabilization. Following systemic optimization, including exchange transfusion, follow-up fluorescein angiography demonstrated a reduction in peripheral retinal nonperfusion in both patients without progression of neovascularization. These findings suggest that retinal perfusion in SCR may remain dynamic following systemic optimization and raise questions regarding the optimal timing of local retinal treatment. Prospective quantitative studies are needed to further characterize these changes and their potential implications for management.

## Introduction

Sickle cell retinopathy (SCR) is a microvascular complication of sickle cell disease caused by recurrent vaso-occlusive events and chronic hypoxia, leading to progressive peripheral retinal ischemia and, in advanced stages, ischemia-driven neovascularization [1-3]. The clinical spectrum of SCR is classically described using Goldberg’s classification, which continues to guide management strategies ranging from observation in early non-proliferative stages to laser photocoagulation in proliferative disease [4]. However, the natural history of SCR is heterogeneous, and retinal vascular changes may not always be static or irreversible. Several studies have shown that retinal ischemia may stabilize, regress, or progress over time and may be influenced by systemic factors such as hemoglobin composition and hematologic management [3,5-7]. Increasing evidence also suggests that exchange transfusion and hydroxyurea therapy may contribute to retinal stabilization and, in selected cases, improved retinal perfusion [6-9].

The interaction between systemic optimization and retinal perfusion remains incompletely understood, raising questions regarding the optimal timing of local retinal treatment in patients presenting with severe systemic vaso-occlusive disease.

We report two patients with SCR who demonstrated an unexpected angiographic reduction in peripheral retinal nonperfusion following systemic optimization, highlighting the potentially dynamic nature of retinal perfusion and the importance of close collaboration between ophthalmologists and hematologists in individualizing treatment decisions.

## Case presentation

Case 1

A 34-year-old woman with known homozygous sickle cell disease (HbSS) presented on Day 1, with a three-day history of decreased vision in the left eye during hospitalization for a severe vaso-occlusive crisis. She reported previous retinal laser treatment several years ago, without available records, and no subsequent ophthalmologic follow-up.

On initial examination, best-corrected visual acuity (BCVA) was 20/20 in the right eye and counting fingers (CF) in the left eye. Intraocular pressure (IOP) and anterior segment examination were unremarkable bilaterally.

Posterior segment examination revealed peripheral retinal vascular abnormalities consistent with SCR. Ultra-widefield color fundus photography (Figure 1), fluorescein angiography (FA), and macular optical coherence tomography (OCT) were performed.

**Figure 1:**
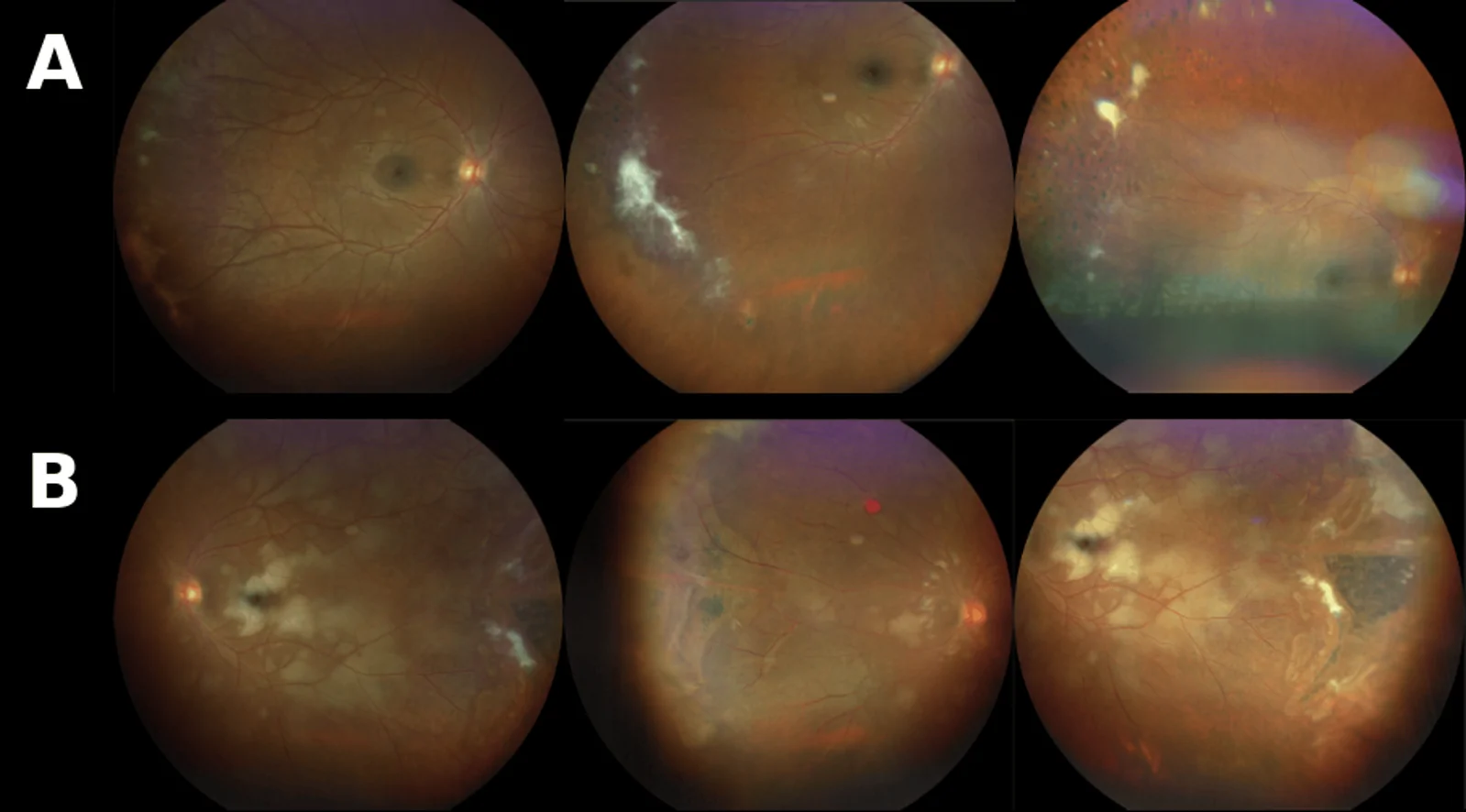
Baseline Color Fundus Photographs - Case 1 Color fundus photographs were obtained on Day 1 using the ZEISS CLARUS 700 (Carl Zeiss Meditec AG, Jena, Germany). (A) Right eye showing peripheral retinal pigmentation consistent with previous laser photocoagulation and fibrotic neovascular proliferation. (B) Left eye showing a swollen, pale macula, a salmon-patch hemorrhage, and a large black sunburst lesion in the temporal retina.

FA of the right eye (Figure 2A) demonstrated peripheral arteriolar occlusions, extensive capillary nonperfusion, incomplete previous laser photocoagulation, and peripheral sea-fan neovascularization, consistent with Goldberg stage III proliferative SCR [4]. FA of the left eye (Figure 2B) revealed extensive peripheral retinal ischemia, markedly more severe than in the right eye, with associated macular ischemia but no evidence of neovascularization, consistent with Goldberg stage II SCR [4].

**Figure 2:**
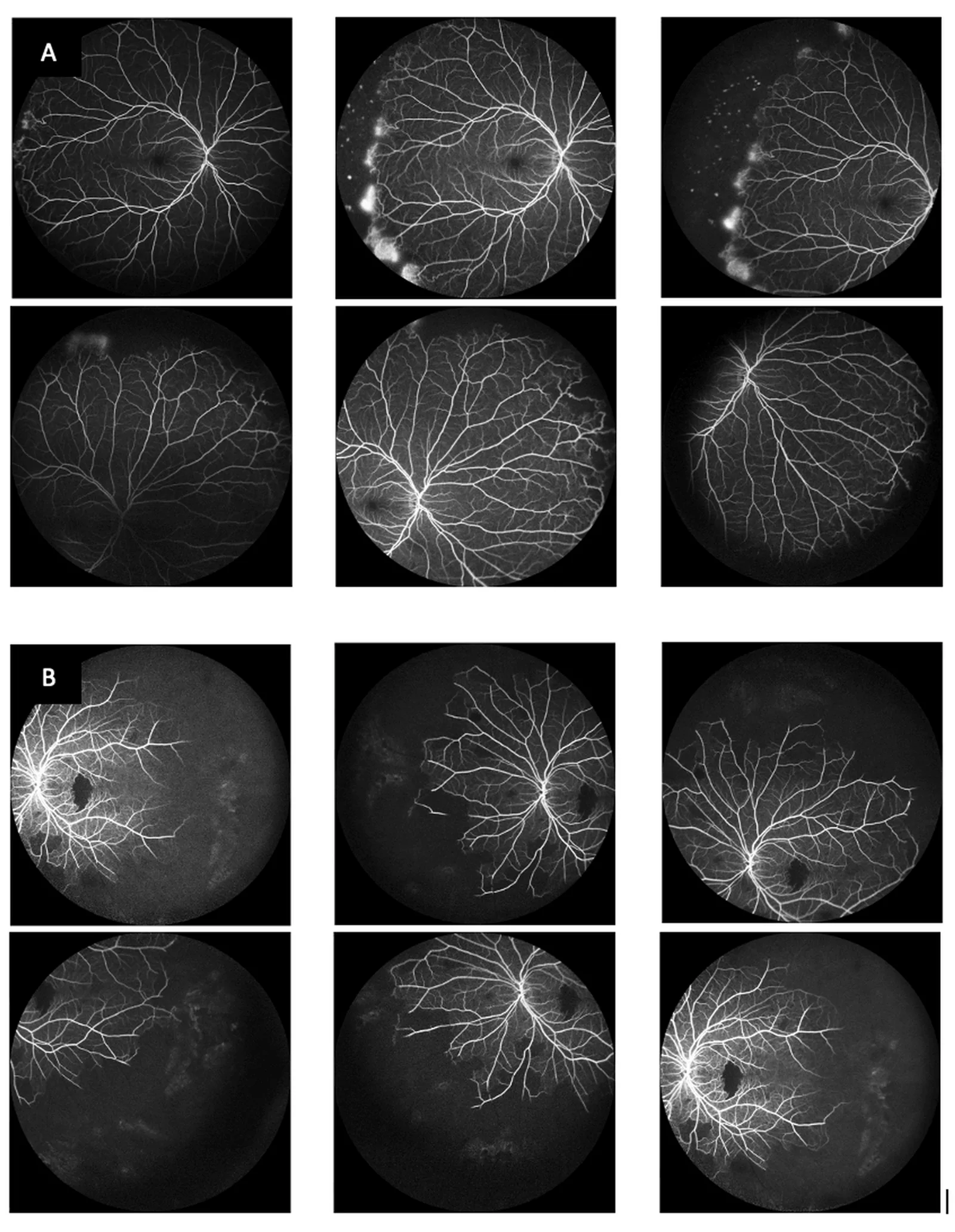
Baseline Ultra-Widefield Fluorescein Angiography - Case 1 Ultra-widefield fluorescein angiography performed on Day 1 using the Heidelberg Spectralis (Heidelberg Engineering GmbH, Heidelberg, Germany). (A) Right eye showing peripheral arteriolar occlusions, extensive capillary nonperfusion, incomplete previous laser photocoagulation, and peripheral sea-fan neovascularization, consistent with Goldberg stage III proliferative SCR. (B) Left eye showing extensive peripheral retinal nonperfusion, markedly more severe than in the right eye, with associated macular ischemia but no evidence of neovascularization, consistent with Goldberg stage II SCR. SCR: sickle cell retinopathy

Macular OCT showed no significant abnormalities in the right eye. In contrast, OCT of the left eye (Figure 3) demonstrated hyperreflective thickening of the inner retinal layers, consistent with acute ischemic changes, as well as marked retinal thinning in the temporal macula.

**Figure 3:**
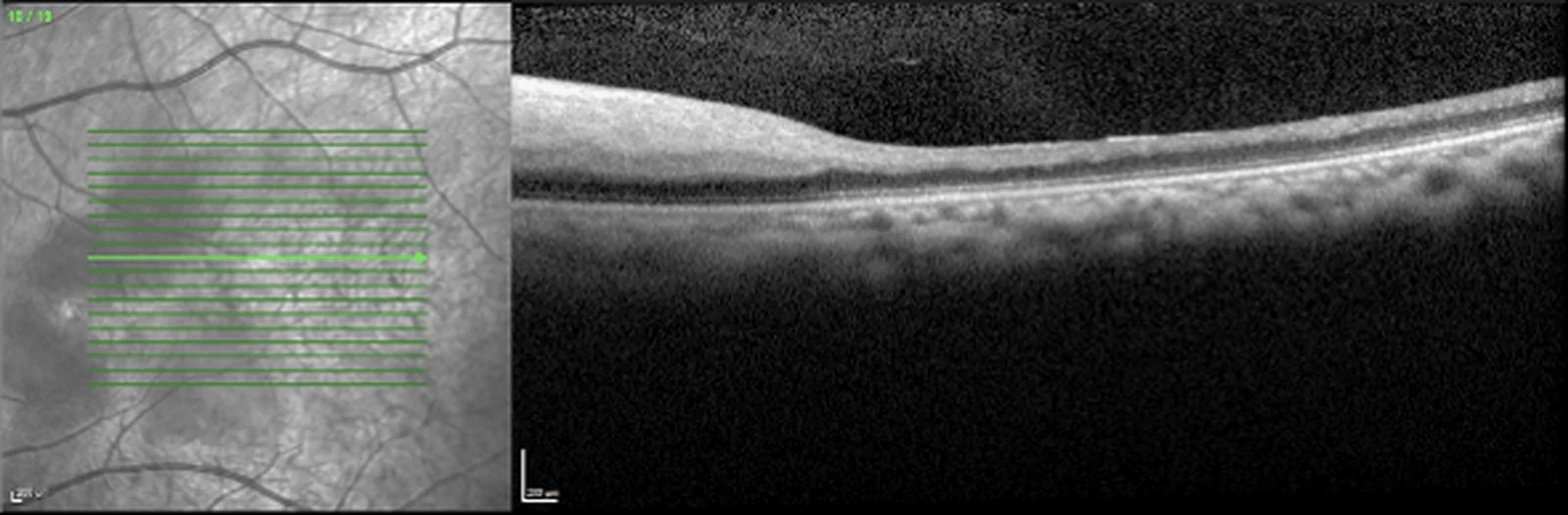
Baseline Macular OCT of the Left Eye - Case 1 OCT images obtained on Day 1 using the Heidelberg Spectralis (Heidelberg Engineering GmbH, Heidelberg, Germany), showing hyperreflective thickening of the inner retinal layers consistent with acute ischemic changes, accompanied by temporal macular thinning. OCT: optical coherence tomography

Given the bilateral extent of peripheral ischemia and the presence of proliferative disease in the right eye, sectoral laser photocoagulation targeting areas of peripheral retinal nonperfusion was performed in both eyes over four sessions during the following weeks. The left eye, although classified as Goldberg stage II without neovascularization [4], was treated because of the extensive peripheral ischemia observed during the acute crisis.

In parallel, during the same hospitalization, the systemic vaso-occlusive crisis was complicated by acute chest syndrome and bilateral pneumonia. Following exchange transfusion and optimization of hydroxyurea therapy, hemoglobin increased from 10.1 to 11.0 g/dL, and the proportion of sickle hemoglobin (HbS) decreased from 49% to 29%.

Although a follow-up visit had been scheduled, the patient returned on Day 170, approximately five months later, during a milder vaso-occlusive crisis. She reported intermittent visual disturbances in the right eye, described as small “dots,” predominantly noticeable in the morning for seven to 10 days. BCVA was 20/20 in the right eye and 20/400 in the left eye. IOP and slit-lamp examination remained normal.

Follow-up FA of the left eye (Figure 4) demonstrated a marked reduction in angiographically detectable peripheral nonperfusion, suggestive of partial retinal reperfusion, with no evidence of neovascularization or new hemorrhagic complications. Both baseline and follow-up images corresponded to the intermediate phase of the angiogram, allowing reasonable comparison of peripheral perfusion. OCT of the left eye (Figure 5) showed resolution of inner retinal edema with residual retinal thinning.

**Figure 4:**
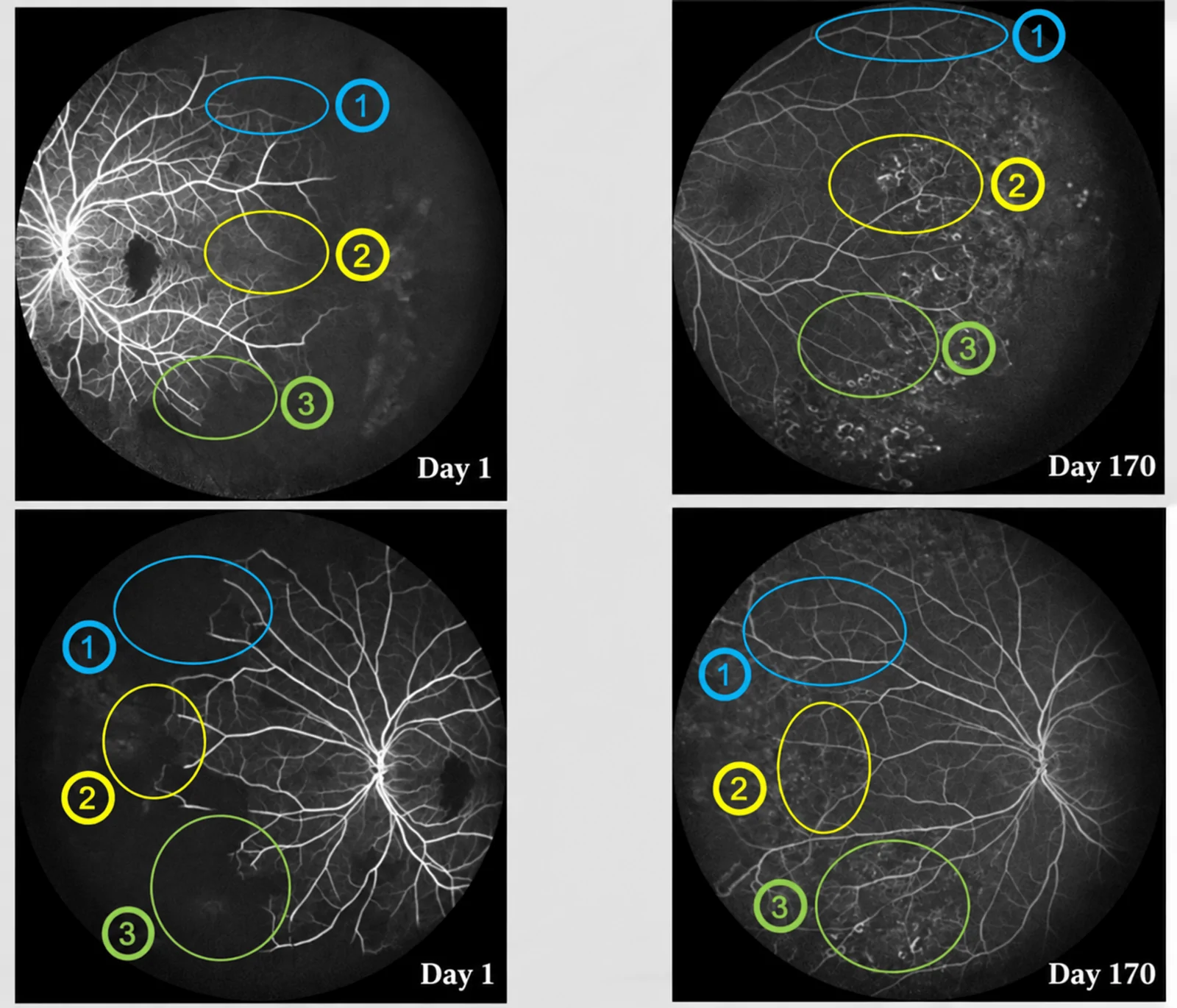
Baseline and Follow-Up Ultra-Widefield Fluorescein Angiography of the Left Eye - Case 1 Ultra-widefield fluorescein angiography images acquired using the Heidelberg Spectralis (Heidelberg Engineering GmbH, Heidelberg, Germany) at baseline (Day 1; 03:05 min, left panels) and follow-up (Day 170; 03:45 min, right panels). Labels 1, 2, and 3 identify corresponding peripheral retinal sectors at baseline and follow-up, demonstrating increased angiographically visible vascular perfusion and a reduction in areas of nonperfusion.

**Figure 5:**
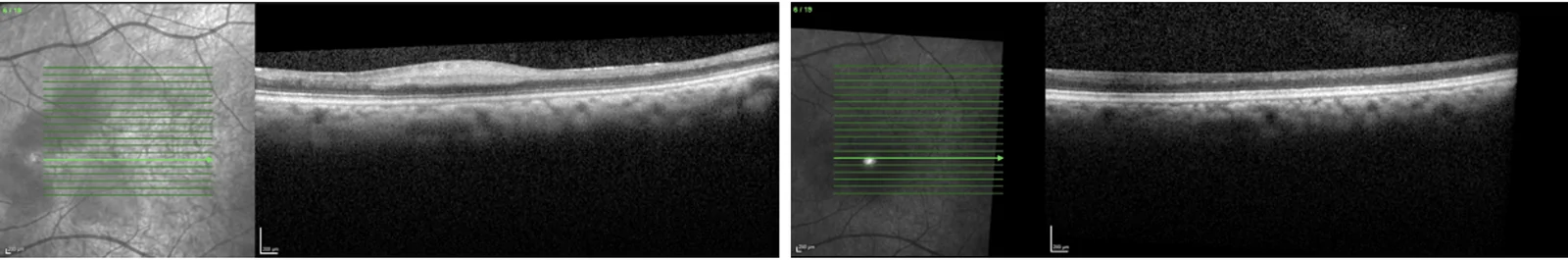
Baseline and Follow-Up Macular OCT of the Left Eye - Case 1 Macular OCT images acquired using the Heidelberg Spectralis (Heidelberg Engineering GmbH, Heidelberg, Germany). Left: Baseline OCT obtained on Day 1, showing hyperreflective thickening of the inner retinal layers consistent with acute ischemic changes. Right: Follow-up OCT obtained on Day 170, showing resolution of inner retinal edema with residual retinal thinning. OCT: optical coherence tomography

Case 2

A 15-year-old boy with known HbSS, receiving long-term hydroxyurea therapy for recurrent vaso-occlusive crises, presented with a three-day history of progressive visual loss in the right eye associated with pain on eye movements.

On Day 1, initial examination showed a BCVA of CF in the right eye and 20/20 in the left eye. Fundus examination (Figure 6) revealed optic disc edema and marked vascular tortuosity in the right eye. OCT of the right eye (Figure 7) demonstrated paracentral acute middle maculopathy (PAMM). FA of the right eye (Figure 8) demonstrated severely delayed retinal perfusion with extensive peripheral nonperfusion.

**Figure 6:**
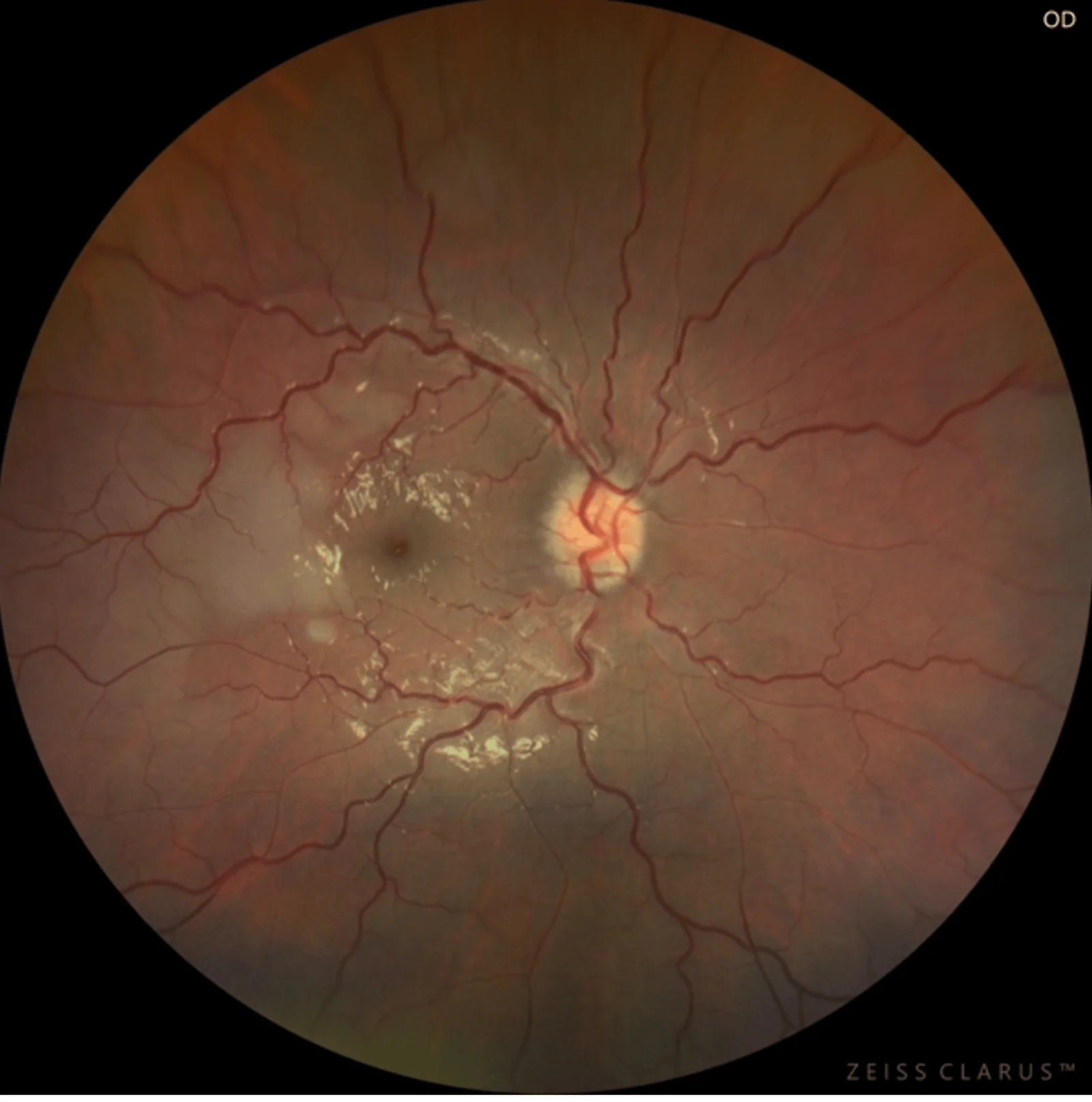
Baseline Color Fundus Photograph of the Right Eye - Case 2 Color fundus photograph obtained on Day 1 using the ZEISS CLARUS 700 (Carl Zeiss Meditec AG, Jena, Germany), showing optic disc edema, temporal retinal edema involving the macula, diffuse superior retinal edema, and marked vascular tortuosity.

**Figure 7:**
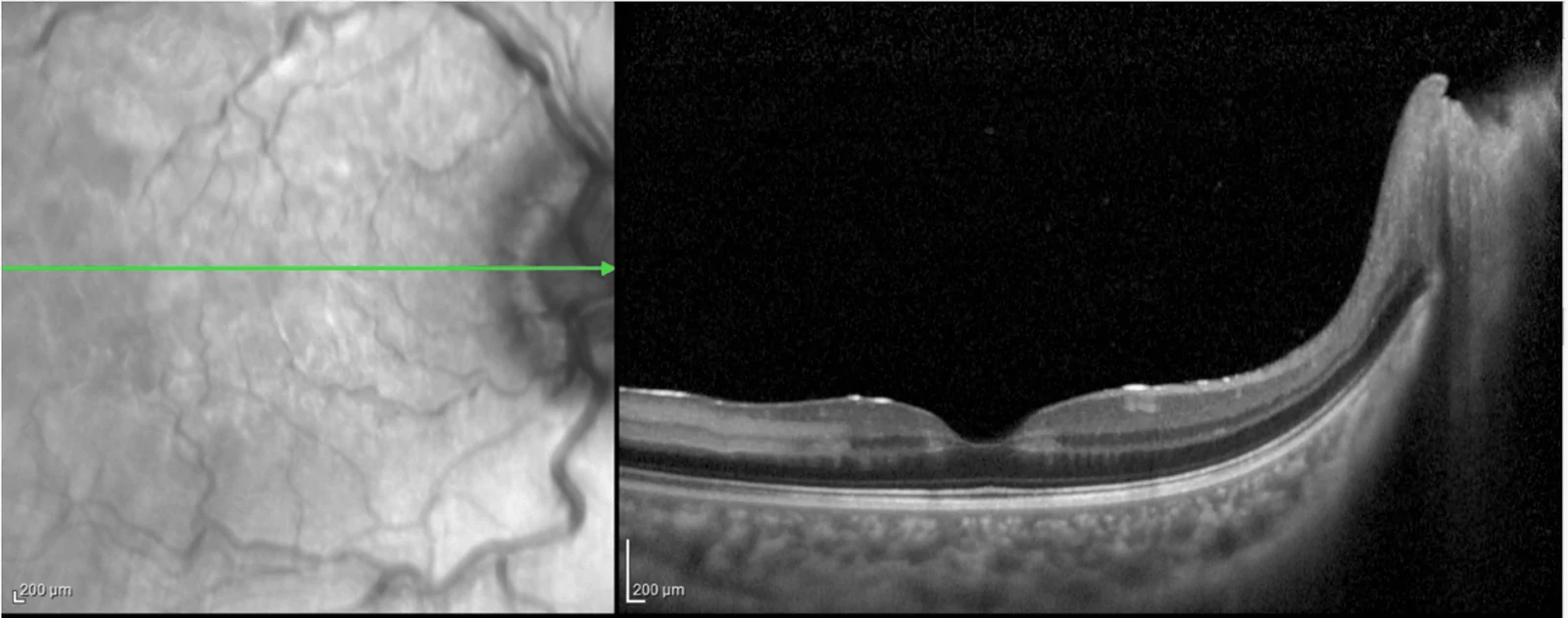
Baseline Macular OCT of the Right Eye - Case 2 Macular OCT images obtained on Day 1 using the Heidelberg Spectralis (Heidelberg Engineering GmbH, Heidelberg, Germany), showing optic disc edema and hyperreflectivity of the inner retinal layers temporal to the fovea, consistent with paracentral acute middle maculopathy (PAMM). OCT: optical coherence tomography

**Figure 8:**
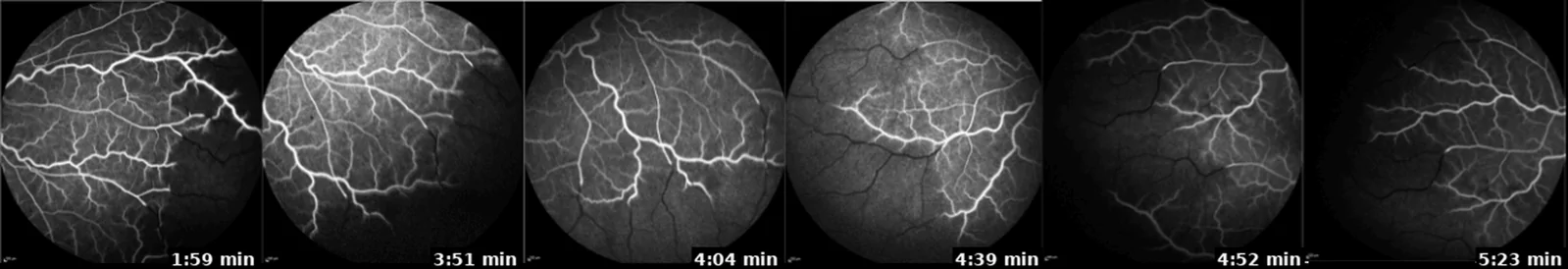
Baseline Ultra-Widefield Fluorescein Angiography of the Right Eye - Case 2 Ultra-widefield fluorescein angiography images obtained on Day 1 using the Heidelberg Spectralis (Heidelberg Engineering GmbH, Heidelberg, Germany), showing extensive peripheral retinal capillary nonperfusion involving multiple quadrants, predominantly the nasal, inferior, and temporal retina. Representative images acquired during the intermediate phase of the angiogram are shown. The specific time point after fluorescein injection is indicated on each image.

Laboratory investigations showed chronic hemolysis with elevated bilirubin and reticulocytosis, as well as neutrophilic leukocytosis (Table 1).

**Table 1:** Laboratory Findings at Initial Presentation (Day 1) - Case 2 INR: international normalized ratio

Laboratory Parameter	Result	Reference Range
Hemoglobin	10.5 g/dL	13.0-16.0 g/dL
Red blood cell count	3.65 × 10^6^/µL	4.5-5.5 × 10^6^/µL
Hematocrit	29.6%	37-49%
White blood cell count	13.46 × 10^3^/µL	4.0-10.0 × 10^3^/µL
Neutrophil count	10.58 × 10^3^/µL	1.5-7.5 × 10^3^/µL
Neutrophils	78.6%	40-75%
Lymphocytes	10.8%	20-45%
Reticulocyte count	140.9 × 10^3^/µL	25-100 × 10^3^/µL
Reticulocytes	3.86%	0.5-2.5%
Prothrombin time	66%	70-100%
INR	1.3	0.8-1.2
Creatinine	48.68 µmol/L	60-110 µmol/L
Total bilirubin	3.27 mg/dL	0.2-1.2 mg/dL
Direct bilirubin	0.38 mg/dL	<0.3 mg/dL
Hypochromic red blood cells	2.5%	<2.5%

Brain and orbital magnetic resonance imaging showed swelling and contrast enhancement of the prechiasmatic right optic nerve. Based on the clinical and imaging findings, an acute optic neuropathy with a presumed ischemic component was considered. The patient was promptly treated with intravenous hyperhydration, an intravitreal anti-vascular endothelial growth factor (VEGF) injection, and exchange transfusion before transfer to our tertiary pediatric intensive care unit.

On Day 4, BCVA remained CF in the right eye. A second exchange transfusion reduced HbS from >80% to <30%. Persistent hemolysis and fluctuating inflammatory markers were observed, with a transient CRP peak of approximately 78 mg/L. Extensive infectious and immunological investigations, including cerebrospinal fluid analysis, were unrevealing.

Although the overall presentation favored an ischemic mechanism, persistent ocular pain and optic nerve enhancement on MRI raised suspicion of a concomitant inflammatory optic neuropathy. After exclusion of infectious causes, high-dose intravenous methylprednisolone was administered for five days.

From Day 7 onward, BCVA progressively improved, reaching 20/200 initially, 20/100 by Day 10, and 20/40 by Day 16. This improvement was accompanied by a marked reduction in retinal edema on OCT and decreased vascular tortuosity. By Day 24, optic disc edema had nearly resolved, with no evidence of neovascularization. At Day 55, BCVA remained stable at 20/40, with a residual visual field defect and mild optic disc pallor.

Follow-up FA performed on Day 55 (Figure 9) demonstrated a marked reduction in angiographically detectable peripheral nonperfusion, suggestive of partial retinal reperfusion, without evidence of neovascularization. The patient subsequently underwent monthly exchange transfusions for three months, with no recurrence of acute ischemic or inflammatory events.

**Figure 9:**
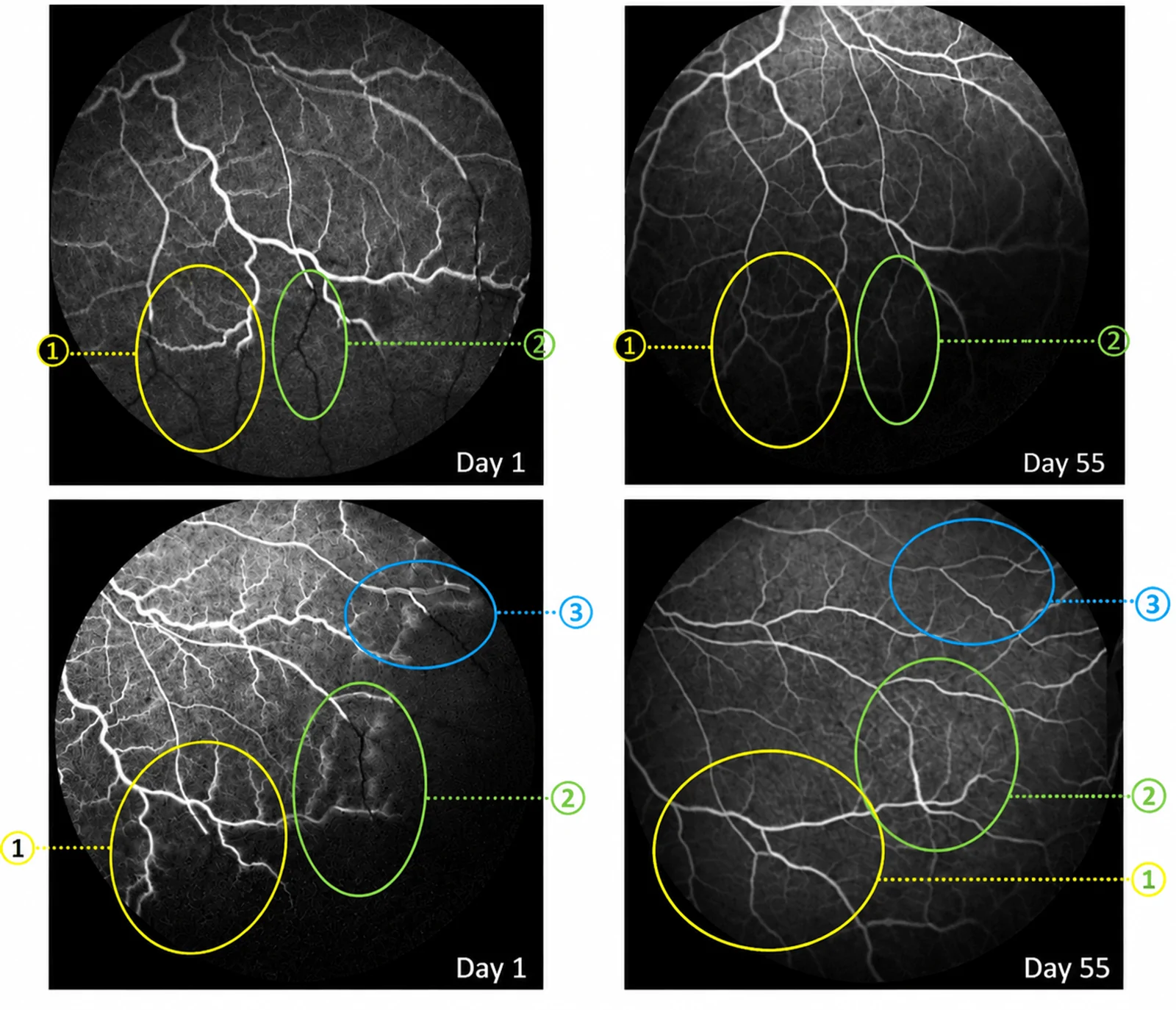
Baseline and Follow-Up Ultra-Widefield Fluorescein Angiography of the Right Eye - Case 2 Ultra-widefield fluorescein angiography images acquired using the Heidelberg Spectralis (Heidelberg Engineering GmbH, Heidelberg, Germany) at baseline (Day 1; left panels) and follow-up (Day 55; right panels). Labels 1, 2, and 3 identify corresponding peripheral retinal sectors at baseline and follow-up, demonstrating increased angiographically visible vascular perfusion and a reduction in areas of nonperfusion. Follow-up angiography shows a marked reduction in angiographically detectable peripheral nonperfusion, suggestive of partial retinal reperfusion, without evidence of neovascularization.

## Discussion

This two-case series describes unexpected angiographic improvement in peripheral retinal perfusion following systemic stabilization in patients with SCR presenting during severe vaso-occlusive crises. In both cases, follow-up FA demonstrated a marked reduction in peripheral capillary nonperfusion, suggestive of partial retinal reperfusion.

Laser photocoagulation remains the standard local treatment for proliferative SCR, primarily targeting neovascularization and preventing complications such as vitreous hemorrhage and tractional retinal detachment [3,10]. However, laser addresses the downstream angiogenic consequences of retinal ischemia rather than the underlying vaso-occlusive process. Evidence supporting its efficacy remains limited, with the most recent Cochrane review rating the certainty of evidence as low to very low [10]. Our observations do not challenge the role of laser in established proliferative disease but raise the possibility that treatment timing may warrant consideration in selected patients presenting during severe systemic vaso-occlusive crises.

The natural history of SCR is heterogeneous, with retinal vascular lesions capable of stabilizing, regressing, or progressing over time. Proliferative sea-fan lesions may also undergo spontaneous auto-infarction [3,5], supporting the concept of retinal vascular remodeling as a dynamic rather than irreversible process. A previous case report in Ophthalmology Retina described spontaneous reversal of peripheral retinal nonperfusion in SCR, providing a clinical precedent for the angiographic changes observed in our cases [11].

This is consistent with sequential OCT angiography findings reported by Zhou et al., who demonstrated intermittent retinal capillary perfusion, with vessels appearing perfused on one scan and occluded on another [12]. Our ultra-widefield angiographic observations may extend this concept to the peripheral retina and suggest that at least some areas interpreted as nonperfused may represent intermittently or sluggishly perfused vascular beds capable of recovery.

In both patients, angiographic improvement followed systemic stabilization and occurred in association with exchange transfusion and hydroxyurea therapy. Exchange transfusion reduces the proportion of HbS-containing erythrocytes and improves oxygen-carrying capacity, while hydroxyurea increases fetal hemoglobin (HbF) and reduces erythrocyte sickling [6,13]. Higher HbF levels have been associated with a lower prevalence and severity of SCR [7]. Previous reports have also described stabilization of proliferative SCR with chronic red cell exchange and reversal of paracentral occlusive retinopathy following exchange transfusion [8,9]. Together, these observations support a potential influence of systemic hematologic optimization on retinal vascular changes.

The temporal relationship observed in our cases is particularly notable. Initial angiography was performed during severe vaso-occlusive crises, whereas improvement became apparent after systemic treatment and stabilization. Whether this represents true reperfusion of previously nonperfused capillary beds, recruitment of partially perfused microvascular networks, or improved visualization of residual circulation cannot be determined from these cases alone. Nevertheless, the observed changes suggest that retinal perfusion may remain dynamic beyond the acute vaso-occlusive phase.

In the second patient, PAMM provided additional evidence of retinal ischemia. PAMM reflects ischemia involving the intermediate and deep retinal capillary plexuses and has been reported in sickle cell disease [14]. Because FA may not adequately detect abnormalities of the deep capillary plexus, structural OCT and OCT angiography provide complementary information in assessing retinal ischemia [15].

These findings may have implications for the timing of local treatment. Current management generally favors observation in Goldberg stage II disease, with laser photocoagulation reserved for proliferative lesions [3,10]. In selected patients presenting with severe systemic vaso-occlusive disease but without immediate vision-threatening complications, repeat vascular imaging after systemic stabilization may help determine whether peripheral ischemia remains dynamic before extensive laser photocoagulation is undertaken. This approach should not delay treatment in eyes with vitreous hemorrhage, tractional retinal detachment, or active progressive neovascularization.

Several limitations should be acknowledged. The small number of patients precludes generalization, and perfusion changes were assessed qualitatively without quantitative metrics. Moreover, multiple interventions, including exchange transfusion and hydroxyurea therapy in both patients, laser photocoagulation in Case 1, and intravitreal anti-VEGF and corticosteroids in Case 2, make attribution of the observed changes to any single treatment difficult. Finally, OCT angiography was not available to corroborate the FA findings.

Prospective studies using serial ultra-widefield FA and OCT angiography, together with detailed hematologic data, are needed to characterize the temporal evolution of retinal perfusion during and after vaso-occlusive crises. Widefield OCT angiography may be particularly valuable because it is non-invasive, repeatable, and quantitative, allowing dynamic perfusion changes to be assessed without the phase dependence of FA [16,17].

## Conclusions

Retinal perfusion in SCR may remain dynamic, and peripheral nonperfusion observed during acute vaso-occlusive crises may not always represent an irreversible endpoint. Our cases suggest that systemic hematologic stabilization may be associated with improvement in retinal perfusion. In selected patients without immediate vision-threatening proliferative complications, repeat vascular imaging after systemic stabilization may help inform the timing of local laser treatment. These observations highlight the importance of close collaboration between ophthalmologists and hematologists to integrate retinal findings with systemic disease activity and individualize management. Prospective studies are warranted to better characterize retinal vascular changes following systemic optimization.

## References

[1] Elagouz M, Jyothi S, Gupta B, Sivaprasad S (2010). Sickle cell disease and the eye: old and new concepts. Surv Ophthalmol.

[2] Menaa F, Khan BA, Uzair B, Menaa A (2017). Sickle cell retinopathy: improving care with a multidisciplinary approach. J Multidiscip Healthc.

[3] Amissah-Arthur KN, Mensah E (2018). The past, present and future management of sickle cell retinopathy within an African context. Eye (Lond).

[4] Goldberg MF (1971). Classification and pathogenesis of proliferative sickle retinopathy. Am J Ophthalmol.

[5] Downes SM, Hambleton IR, Chuang EL, Lois N, Serjeant GR, Bird AC (2005). Incidence and natural history of proliferative sickle cell retinopathy: observations from a cohort study. Ophthalmology.

[6] Howard J (2016). Sickle cell disease: when and how to transfuse. Hematology Am Soc Hematol Educ Program.

[7] Estepp JH, Smeltzer MP, Wang WC, Hoehn ME, Hankins JS, Aygun B (2013). Protection from sickle cell retinopathy is associated with elevated HbF levels and hydroxycarbamide use in children. Br J Haematol.

[8] McKinney CM, Siringo F, Olson JL, Capocelli KE, Ambruso DR, Nuss R (2015). Red cell exchange transfusion halts progressive proliferative sickle cell retinopathy in a teenaged patient with hemoglobin SC disease. Pediatr Blood Cancer.

[9] Gustave BW, Oliver SC, Mathias M (2013). Reversal of paracentral occlusive retinopathy in a case of sickle cell disease using exchange transfusion. Ophthalmic Surg Lasers Imaging Retina.

[10] Myint KT, Sahoo S, Thein AW, Moe S, Ni H (2022). Laser therapy for retinopathy in sickle cell disease. Cochrane Database Syst Rev.

[11] Ramtohul P, Minvielle W, Couturier A (2021). Spontaneous reversal of peripheral nonperfusion in sickle cell retinopathy. Ophthalmol Retina.

[12] Zhou DB, Castanos MV, Pinhas A (2021). Quantification of intermittent retinal capillary perfusion in sickle cell disease. Biomed Opt Express.

[13] Charache S, Terrin ML, Moore RD (1995). Effect of hydroxyurea on the frequency of painful crises in sickle cell anemia. Investigators of the multicenter study of hydroxyurea in sickle cell anemia. N Engl J Med.

[14] Ong SS, Ahmed I, Scott AW (2021). Association of acute macular neuroretinopathy or paracentral acute middle maculopathy with sickle cell disease. Ophthalmol Retina.

[15] Hussnain SA, Coady PA, Stoessel KM (2017). Paracentral acute middle maculopathy: precursor to macular thinning in sickle cell retinopathy. BMJ Case Rep.

[16] Bistour A, Mehanna CJ, Chuttarsing B (2023). Widefield oct-angiography-based classification of sickle cell retinopathy. Graefes Arch Clin Exp Ophthalmol.

[17] Clarke K, Mannath A, Anastasi M (2025). Optical coherence tomography angiography as a tool for diagnosis and monitoring of sickle cell related eye disease: a systematic review and meta-analysis. Eye (Lond).

